# Clinical and research applications of fibroblast activation protein-α inhibitor tracers: a review

**DOI:** 10.1093/bjr/tqaf020

**Published:** 2025-02-05

**Authors:** Mélanie Desaulniers, Étienne Rousseau, Kim M Pabst

**Affiliations:** Department of Nuclear Medicine and Radiobiology, Université de Sherbrooke, Sherbrooke, QC, J1H 5H3, Canada; Research Center of CHUS, CIUSSSE—CHUS, Sherbrooke, QC, J1H 5H3, Canada; Department of Nuclear Medicine and Radiobiology, Université de Sherbrooke, Sherbrooke, QC, J1H 5H3, Canada; Research Center of CHUS, CIUSSSE—CHUS, Sherbrooke, QC, J1H 5H3, Canada; Department of Nuclear Medicine, West German Cancer Center, University Hospital Essen, Essen, 45147, Germany; Cancer Consortium partner site Essen/Düsseldorf, DKFZ and University Hospital Essen, Essen, 45147, Germany

**Keywords:** FAPI PET, FAPI radioligand therapy, fibroblast activation protein inhibitor, theranostics

## Abstract

In the last decade, fibroblast activation protein-α inhibitors (FAPIs), which target the cancer-associated fibroblasts of the tumour microenvironment, have become a topic of great interest. In oncology, FAPI PET/CT imaging has repeatedly demonstrated a higher lesion detection rate than other conventional imaging modalities such as CT or ^18^F-FDG PET/CT for several tumours. In some cases, the initial staging and therapeutic management may even change. Some FAPI radioligands may also be labelled with therapeutic radionuclides for theranostic applications. It is thus possible to treat certain metastatic cancers with FAPI radioligand therapy (FAPI-RLT), which is generally well tolerated with little toxicity. Recently, new FAPIs have been developed with the particularity of having a higher binding affinity for the target, which further improves the lesion detection rate on PET/CT and clinical outcomes following FAPI-RLT. This review provides recent updates in the clinical use of FAPI PET/CT and FAPI-RLT and discusses potential emergent applications, including in inflammation imaging.

## Introduction

Fibroblast activation protein-α (FAP) is a type II transmembrane glycoprotein belonging to the dipeptidyl peptidase family that is expressed by activated fibroblasts during a pathological or inflammatory process.^1^ This glycoprotein is highly expressed by cancer-associated fibroblasts (CAFs) present in the tumour microenvironment (TME) and is present in over 90% of epithelial cancers.^2–4^ CAFs play an important role in tumorigenesis through mechanisms such as promoting neovascularization, suppression of immune surveillance, and production of pro-inflammatory cytokines and growth factors, thereby facilitating tumour progression.^1^^,^^5^ Thus, FAP can be leveraged as a biomarker for certain cancers with potential uses for both imaging and radioligand therapy (RLT), also termed a theranostic application. In recent years, a wide variety of fibroblast activation protein-α inhibitors (FAPIs) targeting FAP have been developed for PET/CT imaging (FAPI-PET) and RLT (FAPI-RLT) and demonstrated at least in breast cancer, digestive cancer, pancreatic cancer, and sarcoma.^6–8^ The aim of this review is to provide an overview of updates and future perspectives on the clinical and research applications of FAPI tracers in theranostics.

## Development of FAPIs

Although FAPIs for PET/CT imaging have gained in popularity in recent years, the interest in imaging tumour stroma FAP expression dates many decades. For instance, in the early 1990s, a ^131^I-labelled F19 antibody was developed and used to image colorectal carcinoma metastases in the liver.^3^^,^^9^ In 2009, the first ^125^I-labelled FAPIs targeting the TME were described.^10^ From 2013 onwards, several more selective FAPIs were developed by Jansen et al, whose work would later be leveraged by Lindner and Loktev to synthesize ^68^Ga-labelled analogues that would reignite interest for the target in the nuclear medicine community.^6^^,^^11^^,^^12^

While many analogues have been developed by numerous groups, the most reported on variants include FAPI-04 (^68^Ga, ^90^Y, ^177^Lu) and FAPI-46 (^68^Ga, ^90^Y, ^177^Lu). Because of their structure including a radionuclide chelator, both diagnostic (eg, ^68^Ga) or therapeutic (eg, ^90^Y, ^177^Lu) radionuclides may be used, enabling theranostic applications.

## Normal biodistribution of FAPI PET/CT

**Figure 1.**
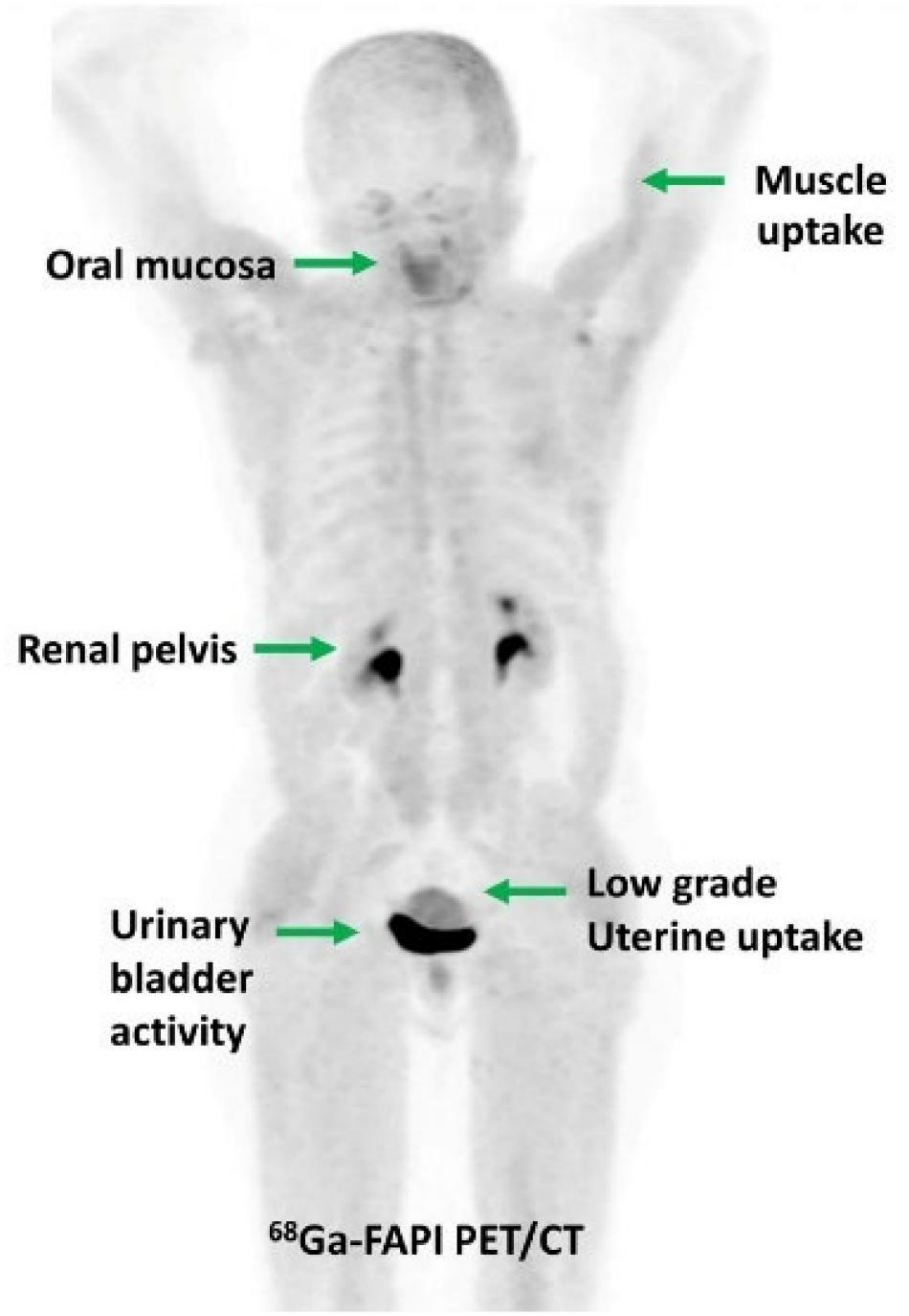
Maximum intensity projection ^68^Ga-FAPI-04 PET demonstrating normal biodistribution in a 55-year-old woman. Image adapted from Chandekar et al.^29^

FAP is not normally expressed in most healthy tissues, in adults, and as such FAPI PET shows little uptake except in the urinary pathway where the tracer is normally excreted (Figure 1).

Nonetheless, some normal tissues can normally show variable uptake on FAPI-PET, including the uterus (Figures 1 and 4), breast, extraocular muscles, salivary glands, tissue surrounding teeth, the nasal oral mucosa, and muscle. The mechanism for such uptake is not completely elucidated and can include both actual FAP expression, as is suspected to be the case for uterine uptake based on tissue expression databases, or potential non-specific uptake for salivary glands, for instance.^14^

## FAPI-PET uptake in inflammation and benign lesions

FAP is not only expressed in CAFs but also in the wider category of activated fibroblasts. These are involved in wound healing and inflammation, and as such, FAPI PET can show uptake in many inflammatory conditions (Table 1), not unlike FDG.

**Table 1.** Common inflammatory/fibrotic conditions with FAPI PET uptake.

**Common inflammatory/fibrotic conditions with FAPI PET uptake** ^29^ ^,^ ^34^ ^,^ ^35^	
Periodontitis	Takayasu’s arteritis
IgG4 disease (salivary, pleural, liver, biliary, pancreas, pituitary, prostate)	Dermatomyositis
Thyroiditis	Juvenile polymyositis
Thermal damage following pulmonary vein isolation	Inflammatory or reactive lymph nodes
Myocarditis	Osteitis
Pneumonia	Scarring
Tuberculosis (lung, liver, intracranial, intestinal, lymph nodes, bone)	Crohn’s disease
Esophagitis	
Cirrhosis	
Pancreatitis	
Renal fibrosis	
Haemorrhoids	
Prostatitis	
Wound healing or inflammation	

**Figure 2.**
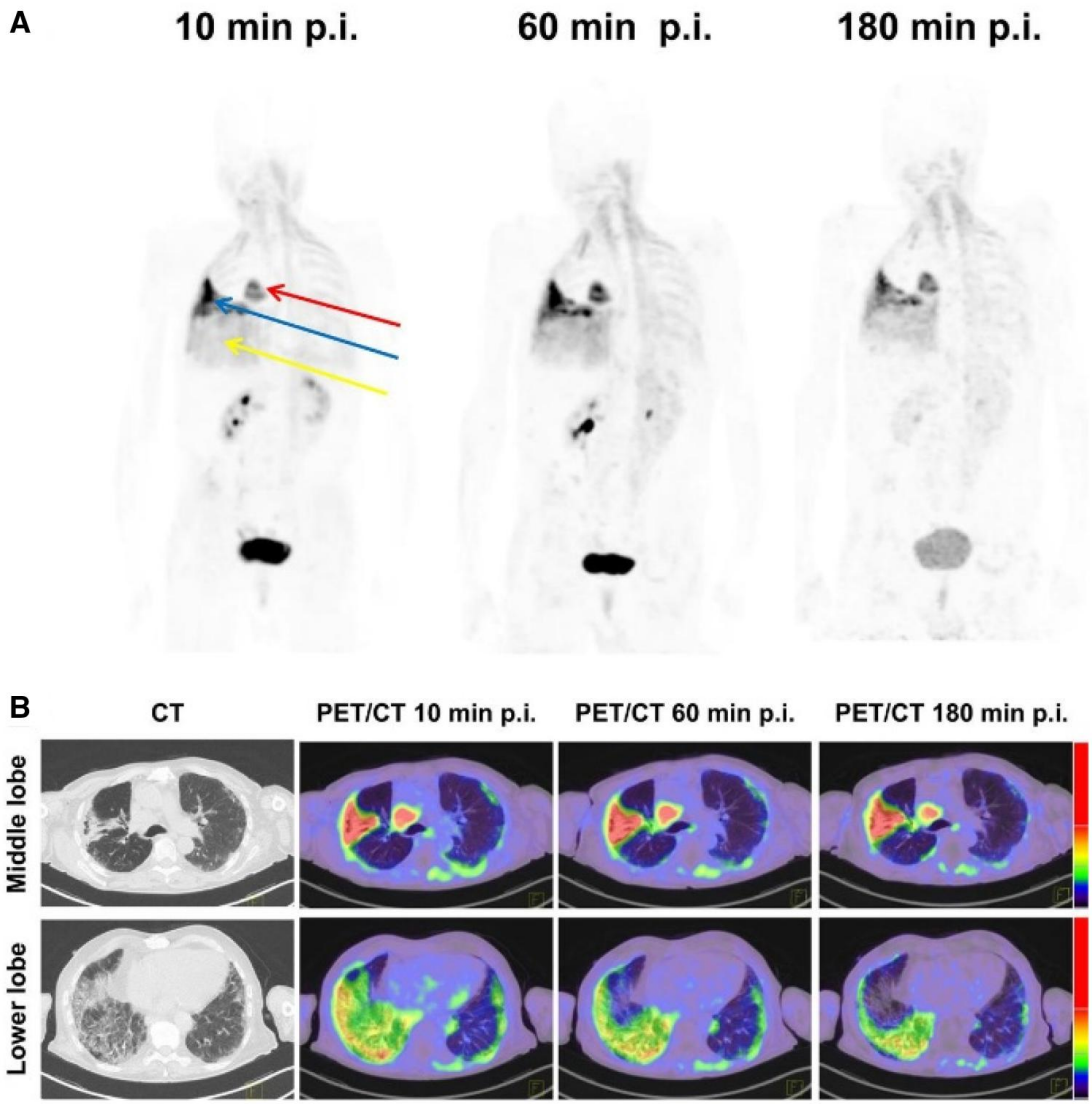
(A) ^68^Ga-FAPI PET maximal intensity projection in a 75-year-old man with rheumatoid arthritis-associated interstitial lung disease (blue and yellow arrows) and non-small-cell lung carcinoma (red arrow). (B) Axial CT images and PET/CT images of pulmonary fibrosis lesions of same patient. The figure was originally published in JNM. Röhrich et al.^15^

**Figure 3.**
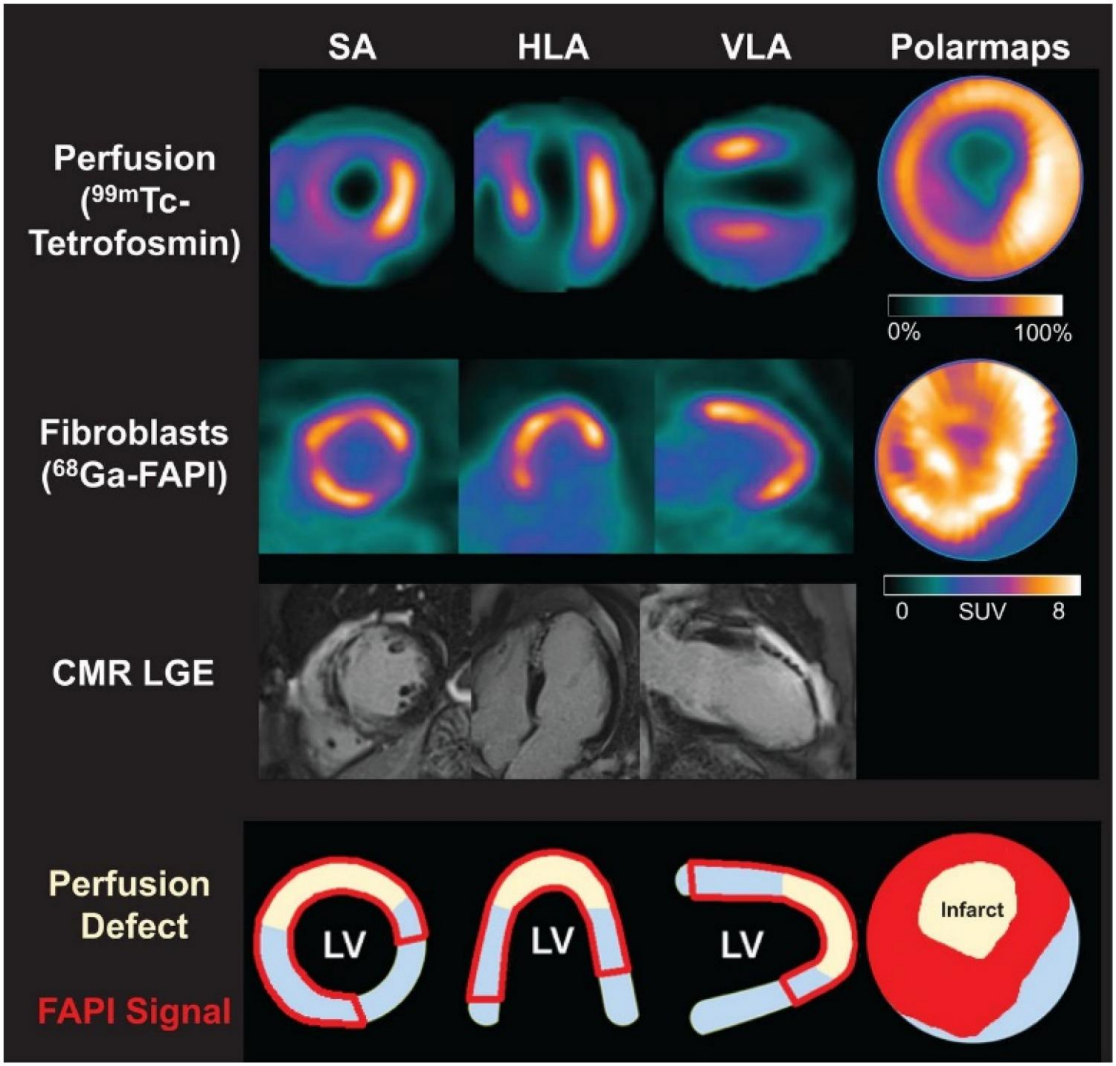
Myocardial perfusion images using ^99m^Tc-tetrofosmin at rest, ^68^Ga-FAPI PET, late gadolinium enhancement (LGE) from cardiac MR, and schematic drawings of left ventricle (LV). Area of fibroblast activation as indicated by ^68^Ga-FAPI-46 PET signal exceeds infarct area and LGE signal, the most common type of myocardial FAP distribution. HLA = horizontal long axis; SA = short axis; VLA = vertical long axis. The figure was originally published in JNM. Figure and legend adapted from Diekmann et al.^16^

Notably, Röhrich et al^15^ demonstrated FAPI accumulation in fibrotic interstitial lung diseases that correlated with CT-based fibrosis index and confirmed FAP expression by immunohistochemistry on human biopsy samples (Figure 2). FAPI accumulation has also been studied in the setting of myocardial infarction. Diekmann et al^16^ reported that FAPI uptake was larger than SPECT perfusion defects in patients and that it was predictive of ventricular dysfunction (Figure 3). Rheumatoid arthritis was also recently studied with FAPI and FDG by Luo et al. Their group found that 6.1% of FAPI-avid articulations were only detected by FAPI PET and that SUV_max_ of both FDG and FAPI correlated with C-reactive protein levels.^17^ Dorst et al^18^ also imaged a rheumatoid arthritis patient and demonstrated light dose-dependent cell death of synovial fibroblasts targeted FAP photodynamic therapy. Inflammatory, fibrotic, traumatic, and degenerative FAPI uptake was also reported in many more conditions, as reviewed systematically by Bentestuen et al^19^ and reported in Kessler et al.’s pictorial analysis.^13^^,^^19^^,^^20^

## FAPI-PET uptake in cancer

There are currently no official indications for FAPI PET, as no radiopharmaceuticals are currently marketed for clinical use. However, the literature supports use cases for FAPI PET/CT in several different cancers; Table 2 is a non-exhaustive list. They are reported to have a higher tumour-to-background ratio on FAPI PET/CT than on FDG PET/CT. It has also been shown that SUV_max_ on FAPI-PET correlates with tumour grade.^23–25^

**Table 2.** Cancers and malignant presentations with FAPI uptake.

Cancers and malignant presentations with FAPI uptake
Glioblastoma^21^
Head and neck cancers^21^
Lung cancer^21^
Breast cancer^21^
Pancreas cancer^21^
Cholangiocarcinoma^22^
Peritoneal carcinomatosis^21^
Ovarian cancer^21^
Bladder cancer^21^
Prostate cancer^21^
Sarcomas^21^
Oesophageal cancer^21^
Gastric cancer^21^
Colorectal cancer^21^

**Figure 4.**
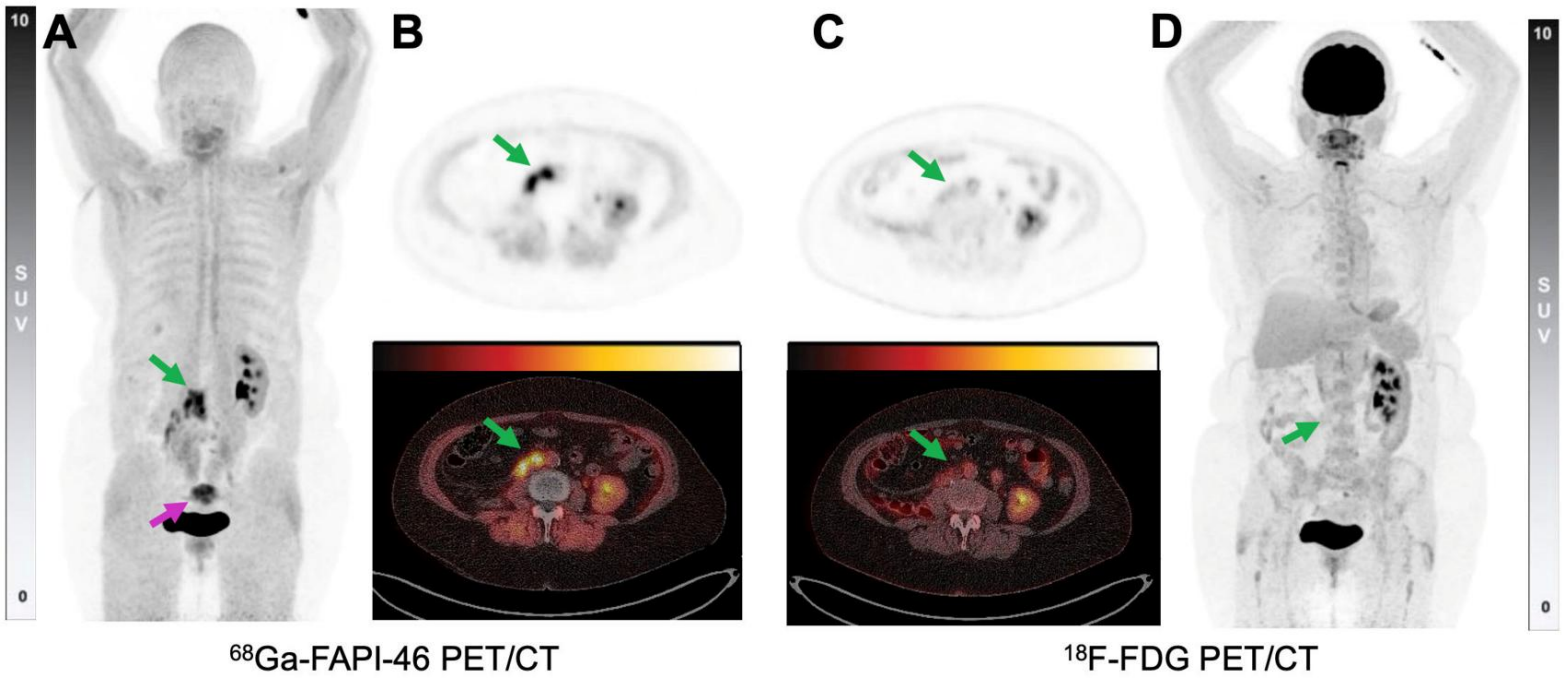
A 63-year-old woman with a local recurrence of a pleomorphic liposarcoma in the right psoas muscle. (A and B) ^68^Ga-FAPI-46 PET/CT shows the local recurrence (green arrow) and physiological intrauterine uptake (pink arrow). (C and D) ^18^F-FDG PET/CT shows the local recurrence (green arrow) as almost non-avid. Maximum intensity projection PET (A and D), axial PET (B and C, top), and axial fusion PET/CT (B and C, bottom). Intrauterine uptake on FAPI PET/CT is a pitfall and tends to decrease after the menopause, although it can be very intense in women of childbearing age.^13^ PET acquisitions were performed on the same day, and the study was performed as part of a basket trial at Universitätsklinikum Essen, Klinik für Nuklearmedizin from the EudraCT 2021-000148-23 trial.

**Figure 5.**
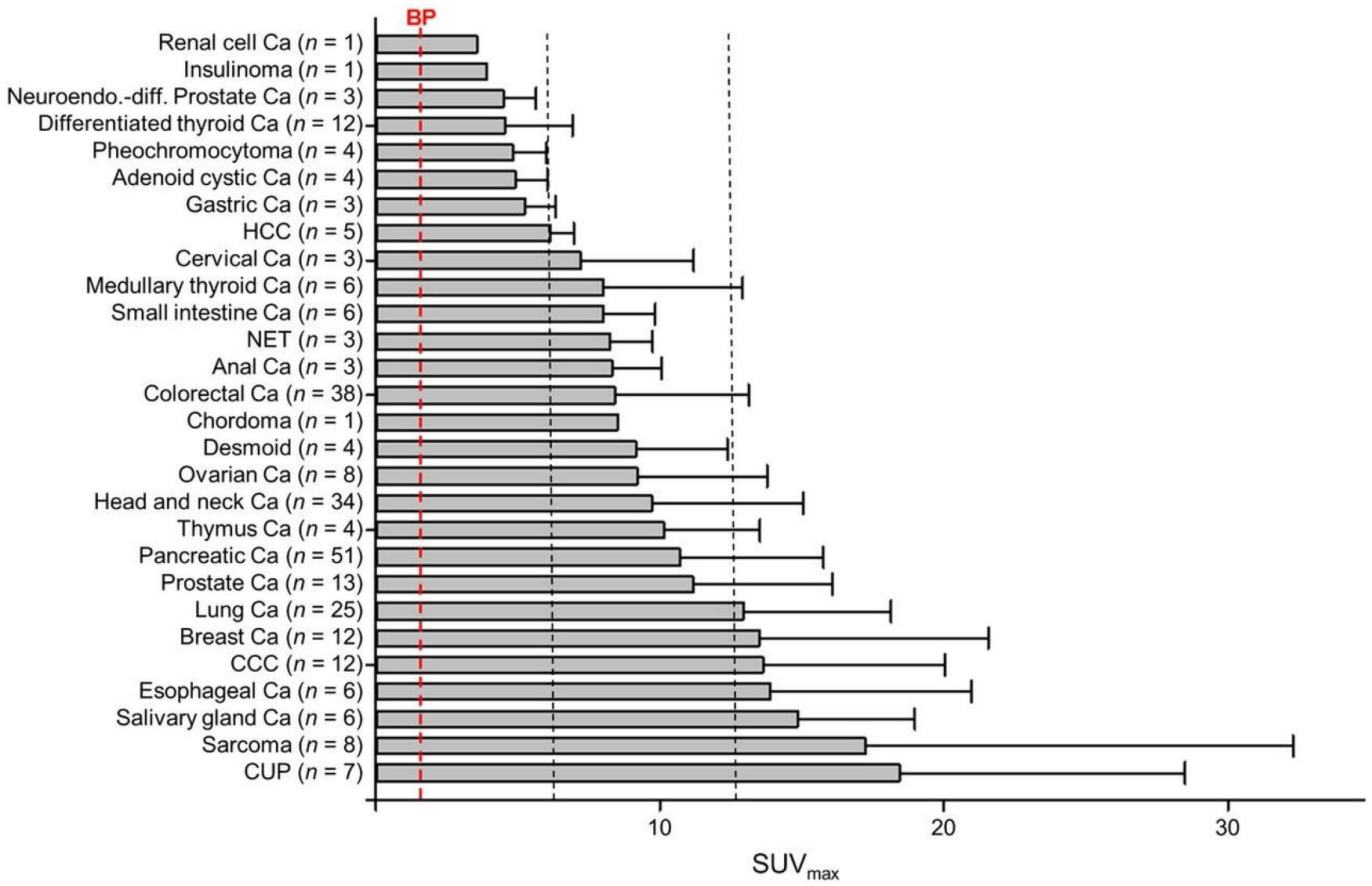
Average SUV_max_ of ^68^Ga-FAPI PET/CT in various tumor entities. Low, intermediate, and high uptake was defined by cutoff at SUVs 6 and 12. By comparison, average background (blood pool) was found to have SUV 1.4. Ca = cancer; CCC = cholangiocellular carcinoma; CUP = carcinoma of unknown primary; HCC = hepatocellular carcinoma; NET = neuroendocrine tumour. The figure was originally published in JNM. Figure and legend from Kratochwil et al.^28^

**Figure 6.**
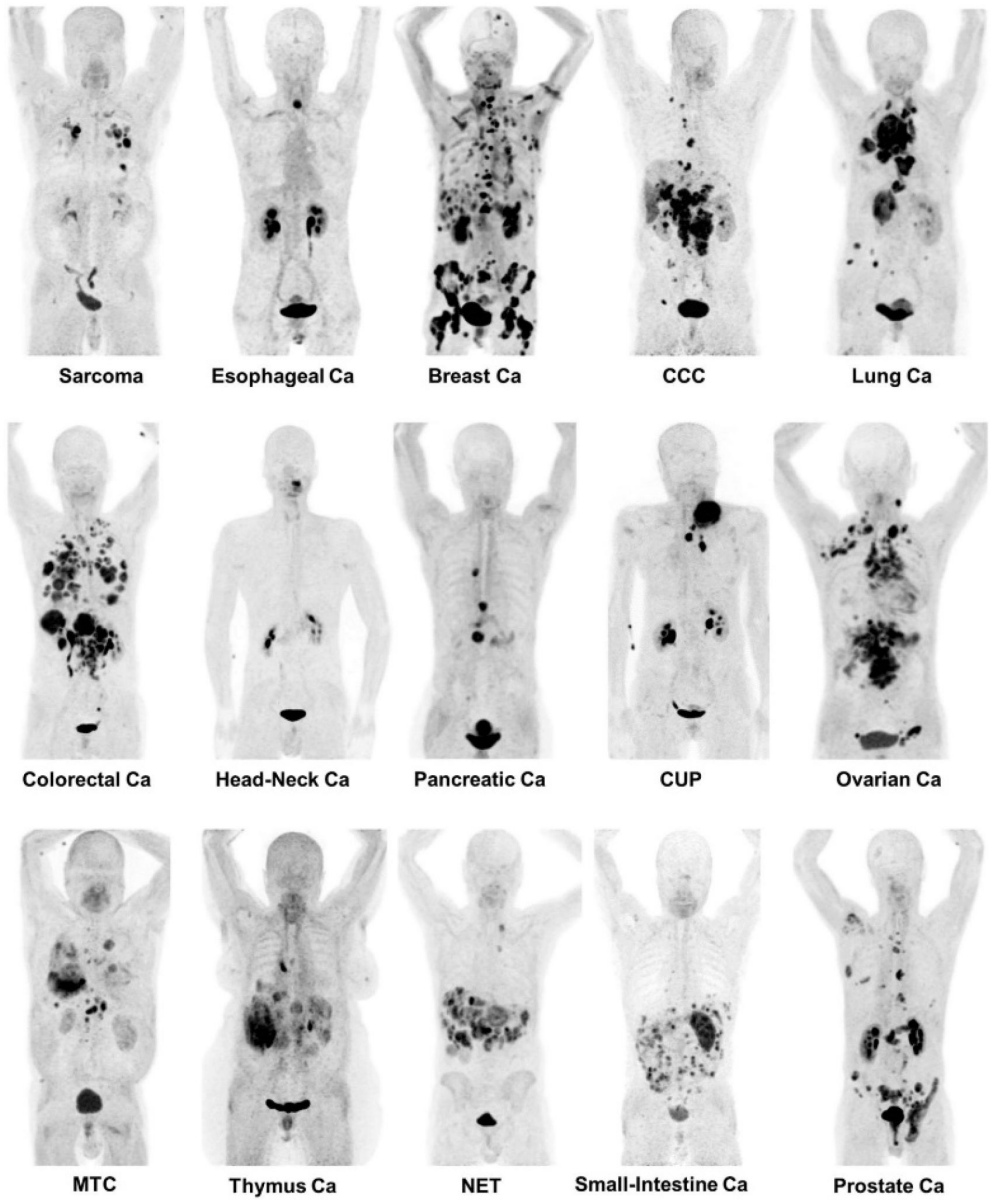
Maximum-intensity projections of ^68^Ga-FAPI PET/CT in patients reflecting 15 different histologically proven tumor entities (sorted by uptake in descending order). Ca = cancer; CCC = cholangiocellular carcinoma; CUP = carcinoma of unknown primary; MTC = medullary thyroid cancer; NET = neuroendocrine tumour. The figure was originally published in JNM. Figure and legend from Kratochwil et al.^28^

FAPI PET/CT seems particularly well suited to imaging tumours where FDG has high physiological uptake or low-tumour uptake. For instance, in head and neck cancers, it could help distinguish residual tumour from post-radiation inflammation, where FDG is hampered by high physiological uptake.^21^ Another indication where the radiotracer is likely to shine is in imaging of sarcoma, where it has been reported superior to FDG^24^^,^^26^^,^^27^; Figure 4 demonstrates this. The breadth of FAPI uptake in cancer was well illustrated by Kratochwil et al’s^28^ work (Figures 5 and 6).

## Will FAPI displace FDG in imaging?

FDG is entrenched as one of the most used tracers in nuclear medicine because it is versatile, being able to image malignancy, inflammation, and infection, relatively cheap and available compared with newer tracers, and, most importantly, well studied.^29^^,^^30^ Notwithstanding not having been approved yet by regulatory agencies for regular clinical use, being a new class of radioligands, it is unlikely that FAPIs will be as available/cheap as FDG in the short term. Furthermore, while more data are available every day, there are still many unknowns about the behaviour of FAPIs in many indications and is expected to take some time before there is sufficient evidence and experience to include it in clinical oncological guidelines.

Recently, several studies have compared FAPI PET/CT with ^18^F-FDG PET/CT in several types of cancer and other inflammatory conditions. Regardless of the clinical indication, what these studies have in common is that FAPI PET/CT contrast is superior to ^18^F-FDG PET/CT (increased lesion activity and very low background), resulting in a very high target-to-background ratio.^31^ An example of this difference in contrast on PET/CT imaging between ^68^Ga-FAPI and ^18^F-FDG is shown in Figure 4.^31^ FAPI may not be always superior to FDG, however. Several studies have demonstrated that some lesions are not picked up by FAPIs. For instance, Civan et al^32^ reported a case of FAPI-negative FDG-positive metastatic breast cancer on PET, Haidar et al^33^ reported false-negative FAPI PET in medullary thyroid carcinoma visible on both ^68^Ga-DOTATOC and ^18^F-FDG PET, and Promteangtrong et al. found that FAPI PET detected less avid nodes than FDG for head and neck squamous cell carcinoma patients. This might be because some tumour subtypes do not recruit many CAFs or that smaller metastases have too small contingents of CAFs to be reliably imaged by that modality, although more research is needed to confirm this.^34^

It is our opinion that FAPIs will initially be a niche tracer used for selected applications where it has consistently demonstrated superiority over FDG, or in selected patients whose tumours are not particularly FDG-avid in addition to FDG, until it becomes cost-effective to use it as a first-line imaging agent.

## Fibroblast activation protein-α inhibitor-radioligand therapy

Since 2020, FAPI-RLT has been used to treat some stage IV cancers that are progressing on an already-approved therapy. Kratochwil et al^35^ published a case report of a patient with fibrous spindle cell soft tissue sarcomas with progressive pulmonary metastases who achieved stable disease for 8 months following 3 cycles of ^153^Sm-FAPI-46 (cumulative dose of 20 GBq) and 1 cycle of ^90^Y-FAPI-46 (8 GBq). As the biological half-life of FAPI-46 (≈ 24 h) is relatively short, ^153^Sm (46.3 h) and ^90^Y (64.1 h) were the radioisotopes of choice as their physical half-life is also short.^35^^,^^36^ Because of the specific activity and ^154^Eu contamination of ^153^Sm, other radioisotopes with short physical half-lives have been studied more closely, such as ^90^Y. Fendler et al treated a cohort of 21 patients with progressive metastatic solid tumours, mainly solitary fibrous tumours, with ^90^Y-FAPI-46.^24^^,^^37–40^ Of these, 16 patients had sarcomas and almost a third of patients had stable disease after FAPI-RLT, which correlated with prolonged overall survival (*P *= .013). ^90^Y-FAPI-46 caused grade 3 or 4 anaemia and thrombocytopenia in only 8 patients and was clinically well tolerated.^37^ Another case report of a patient with breast cancer metastatic to lymph nodes, liver, and bone in addition to colorectal cancer with peritoneal carcinomatosis showed a mixed response to ^90^Y-FAPI-46, that is, an excellent response in the peritoneal carcinomatosis, but with little response in the tumour lesions of the breast cancer.^41^

Despite interesting initial results, tumour retention time was not optimal, and many groups sought to improve this. A few studies have also looked at squaramide-based FAPIs, including dimeric structures. Quinoline-based FAPIs, including FAPI-46, have already shown great potential in therapy, but the relatively short intratumour retention time remains an issue. For an effective FAPI-RLT, the biological half-life of the radiotracer should ideally correspond to the physical half-life of the major therapeutic radionuclides ^177^Lu and ^225^Ac (6.7 and 9.9 d), hence the importance of dimeric FAPIs. Martin et al^42^ optimized DOTAGA.(SA.FAPi)_2_ and developed DOTAGA.Glu.(FAPi)_2_, which has a longer intratumour retention time, binds easily to ^225^Ac, has greater affinity and selectivity, is rapidly excreted, and has less active in the colon. This *in vitro* study showed promising results for ^177^Lu-DOTAGA.Glu.(FAPi)_2_ and ^225^Ac-DOTAGA.Glu.(FAPi)_2_ and the team also performed a proof-of-principle imaging/therapy study in a medullary thyroid cancer patient. Yadav et al^8^ also investigated FAPI dimers in breast cancer patients with 177Lu-DOTAGA-FAPi dimers. They treated 19 patients with 2-6 cycles of 11-33.3 GBq of radioligand, which yielded 25% partial remission. Another approach to lengthening biological half-life was demonstrated by Zboralski et al^43^ with 177Lu-FAPI-2286 that leverages a cyclic peptide structure as binding motif. A clinical trial in 8 patients with metastatic sarcoma that received 4 cycles of 6660-7400 MBq of 177Lu-FAPI-2286 in 6-8 week intervals yielded no grade 3 or 4 adverse effects, demonstrated a 52% reduction in primary tumour volume with concomitant decrease in SUV_max_, and improved overall survival.^44^ Recently, a Chinese team has developed a new tetrameric FAPI, DOTA-4P(FAPI)_4_ and compared it with a dimeric and monomeric FAPI.^45^ Both *in vivo* and *in vitro*, this DOTA-4P(FAPI)_4_ has a higher tumour uptake and a higher intratumour retention time. However, the clearance time is longer than for FAPI dimers and FAPI-46, which results in more background noise on the PET/CT images. With ^177^Lu-DOTA-4P(FAPI)_4_, the tumour suppression tested on mice was remarkable; but there is no clinical data yet.

A number of groups have explored 225Ac therapy at preclinical level. One study showed that the effects of treatment with 177Lu-FAPI-46 were relatively slow but lasted longer than those of 225Ac-FAPI-46.^46^ In most cases, α therapies prove to be more effective than β therapies, with less toxicity for surrounding healthy tissue.^47^ Recently, an American group has been looking at 225Ac-FAPI-46 therapy combined with immunotherapy in immunocompetent mice with sarcomas sensitive or resistant to immunotherapy.^48^ The efficacy of up to three cycles of 60 kBq 225Ac-FAPI-46 in combination with an anti-PD-1 antibody resulted in delayed tumour growth in 55% of mice and partial tumour regression in 18% of mice. These results were superior to 225Ac-FAPI-46 monotherapy; 225Ac-FAPI-46 even appeared to restore sensitivity to immunotherapy in some cases. Thus, while monotherapy has been demonstrated as sufficient to improve patient outcomes, combination therapy might yield even greater benefits. Depletion of TME CAFs, which have an immunosuppressive effect, to improve immunotherapy effectiveness has been an active field of research and preliminary data is encouraging.^49^ There is a strong rationale for such combination therapy with FAPI-RLT.

Despite good results with alpha-emitters in many RLT applications vs beta-emitters, the choice of the ideal radionuclide to accompany FAPI ligands is more complex than with other RLTs that directly target tumour cells. Indeed, FAPI-RLT can have 2 very different targets depending on tumour characteristics. Some tumour cells, particularly in sarcomas, inherently express FAP, while most others express it in the CAFs of their TME. When tumour cells directly express the target, it makes sense to use short-range high linear energy transfer radionuclides, such as alpha emitters like ^225^Ac (47-85 µm range) and Auger electron emitters (nanometre range) to deliver high dose to those cells while sparing adjacent healthy tissues. Those isotopes are usually more effective because they generate more double-stranded DNA breaks that are more lethal.^49^^,^^50^ When the target is the CAFs, alpha and Auger emitters’ short range will not be able to reach actual tumour cells. As stated above, that may potentially be sufficient to deplete TME CAFs and improve immune response. However, to deliver significant radiation dose to actual tumour cells, a longer-range radionuclide, such as ^177^Lu (1.7 mm max range) or ^90^Y (11 mm max range), is required to exploit crossfire effect.^51^ Thus, radionuclides must be chosen wisely based on the target, and on the effect of choice, and there might not be a “one size fits all” approach to FAPI therapy.

FAPI-RLT is still investigational and has not been commercialized yet. As such, accessibility is through clinical trials. It is also used sporadically for compassion in some centres. It is not yet included in oncological algorithms from major societies, mostly because larger phase II/III clinical trials are needed to support their effectiveness. Moreover, the lack of commercial availability and regulatory approval hampers its clinical use.

## Conclusion

Even if relatively new to PET imaging, and FAPI analogues have come a long way in a few years, thanks to the efforts of many research groups who strive to improve the radioligands and document their clinical effectiveness. From initial basket trials, there are now more specific phase II trials that will be able to guide use of the radioligands in clinical practice. It is important to remember that, not unlike FDG, FAPIs are not specific, and that care must be taken in interpreting the images. They show another facet of disease in cancer and inflammation and while they may eventually supersede FDG for some indications, they may also become complimentary radiotracers that helps clinicians draw a more complete portrait of disease, and thus decide on the appropriate care. Going further than FDG, many FAPI analogues can be used for RLT, and while research is still in early stages, it is promising.
